# Hypersensitivity to glucorticoids in children

**DOI:** 10.1186/2045-7022-4-S3-P143

**Published:** 2014-07-18

**Authors:** Tina Vesel, Mira Šilar, Meta Accetto, Uroš Krivec, Simona Žitnik, Vesna Glavnik, Anja Koren Jeverica, Mitja Košnik, Peter Korošec, Tadej Avèin

**Affiliations:** 1 University Children's Hospital, University Medical Center, Department of Allergology, Rheumatology and Clinic, Slovenia; 2 University Clinic of Respiratory and Allergic Diseases, Laboratory for Clinical Immunology and Molecular, Slovenia; 3 University Children's Hospital, University Medical Center, Ljubljana, Department of Pulmonology, Slovenia; 4 University Clinic of Respiratory and Allergic Diseases, Department of Allergology, Slovenia

## Introduction

Immediate hypersensitivity to glucocorticoids is rarely reported, especially in children. Assessment of cross-reactivity after immediate hypersensitivity to intravenous methylprednisolone (MP) usually shows a tolerance to at least one of the alternatives, e.g. MP tablet, hydrocortisone (HC) or dexamethasone (DX).

## Methods

We present 8 children with suspected hypersensitivity reaction after parenteral MP (6 children), DX inhalation (1 child) and inhafluticasone propionate (FL) (1 child). Prick and intradermal skin testing with MP, HC, DX and FL were performed. In case of negative skin tests provocation tests were performed. Basophil activation test (flow cytometry analyses of basophil CD63 surface expression induced by different concentrations of MP, HC, DX and FL (333, 33,3 and 3,33 mcg/ml)) was performed in all patients and in ten controls who tolerated those drugs. Stimulation index (SI) >2 was considered positive.

## Results

All children (5 boys, 3 girls) were atopics and were exposed to various glucocorticoids before systemic hypersensitivity reaction. In 7 children allergy to culprit glucocorticoid was confirmed. 6 children had positive results of either skin testis or provocation test with three or four glucocorticoids and 2 children to MP only. Four children had also positive results of either skin testing or provocation test to FL. In three children positive provocation test after negative skin test confirmed allergy to glucocorticoid. BAT was positive in 6 children: 5 MP, 3 HC, 5 DX, 3 FL. One child with positive skin test to MP had negative BAT. One child was BAT non-responder. One child who tolerated FL had positive BAT to FL. BAT to MP, DX, HC was negative in all ten controls (SI<1). However, BAT with nasal drops of FL was positive in 3 controls (2<si).

